# Treatment of locally advanced carcinomas of head and neck with intensity-modulated radiation therapy (IMRT) in combination with cetuximab and chemotherapy: the REACH protocol

**DOI:** 10.1186/1471-2407-10-651

**Published:** 2010-11-26

**Authors:** Gregor Habl, Alexandra D Jensen, Karin Potthoff, Matthias Uhl, Holger Hof, Jacek Hajda, Christian Simon, Jürgen Debus, Robert Krempien, Marc W Münter

**Affiliations:** 1 Department of Radiation Oncology, University of Heidelberg, INF 400, 69120 Heidelberg, Germany; 2 Department of Radiation Oncology, Helios Klinikum Berlin Buch, Schwanenbecker Chaussee 50, 13125 Berlin, Germany; 3 Department of Otorhinolaryngology, University of Heidelberg, INF 400, 69120 Heidelberg, Germany; 4 Coordination Centre for Clinical Trials (KKS) Heidelberg, University of Heidelberg, Vossstr. 2, 69120 Heidelberg, Germany

## Abstract

**Background:**

Primary treatment of carcinoma of the oro-/hypopharynx or larynx may consist of combined platinum-containing chemoradiotherapy. In order to improve clinical outcome (i.e. local control/overall survival), combined therapy is intensified by the addition of the EGFR inhibitor cetuximab (Erbitux^®^). Radiation therapy (RT) is carried out as intensity-modulated RT (IMRT) to avoid higher grade acute and late toxicity by sparing of surrounding normal tissues.

**Methods/Design:**

The REACH study is a prospective phase II study combining chemoradiotherapy with carboplatin/5-Fluorouracil (5-FU) and the monoclonal epidermal growth factor-receptor (EGFR) antibody cetuximab (Erbitux^®^) as intensity-modulated radiation therapy in patients with locally advanced squamous-cell carcinomas of oropharynx, hypopharynx or larynx.

Patients receive weekly chemotherapy infusions in the 1^st ^and 5^th ^week of RT. Additionally, cetuximab is administered weekly throughout the treatment course. IMRT is delivered as in a classical concomitant boost concept (bid from fraction 16) to a total dose of 69,9 Gy.

**Discussion:**

Primary endpoint of the trial is local-regional control (LRC). Disease-free survival, progression-free survival, overall survival, toxicity, proteomic and genomic analyses are secondary endpoints. The aim is to explore the efficacy as well as the safety and feasibility of this combined radioimmunchemotherapy in order to improve the outcome of patients with advanced head and neck cancer.

**Trial registration:**

ISRCTN87356938

## Background

Long-term outcome for patients with advanced squamous cell carcinoma of the head and neck (SCCHN) is still disappointing. For locally advanced or unresectable SCCHN without evidence of distant metastases, combined chemoradiotherapy is a proven curative treatment option. Due to the sometimes marked side effects in standard chemoradiation regimens using altered-fractionation 3 D conformal radiation techniques, intensification is rarely clinically possible. In view of further improvement of clinical outcome by intensification of the combined treatment approach, toxicity of the established treatment regimen needs to be reduced by employing modern radiotherapy techniques such as IMRT.

A meta-analysis from the MARCH Collaborative Group has shown an absolute survival benefit of 6.5% at 5 years for concurrent chemoradiotherapy [1]. A smaller yet still significant survival benefit was found for all chemoradiotherapy algorithms, whether it was neoadjuvant, adjuvant or concomitant with 4.5% at 5 years [1]. This effect was mainly caused by an increased local control and only to a lesser extent by reduction of distant metastases. The MARCH data showed no difference in response to chemoradiotherapy with respect to tumor site (oral cavity, oro-, hypopharynx, larynx). A published meta-analysis even found an overall survival benefit of 12 months when adding chemotherapy to normally fractionated radiotherapy or altered fractionated schedules [2]. However, no increased benefit was found when using hyperfractionated or accelerated fractionation [conventional fraction of 1.8 or 2.0 Gy per day] in this meta-analysis.

Altered fractionation schedules, especially hyperfractionation, lead to a significant improvement in overall survival if radiation therapy is performed as a single therapy modality. Accelerated radiation therapy alone, however, does not increase overall survival when given as split course or extremely accelerated treatments with decreased total dose. Bourhis et al. came to the same conclusion in their recent meta-analysis: the authors found that altered fractionation schedules showed only a small but again significant, absolute survival benefit when compared to conventional radiotherapy [3]. Also, survival benefit was significantly higher with hyperfractionated radiotherapy than with accelerated radiotherapy. In addition, altered fractionation regimen resulted in increased locoregional control in all patients though younger patients seemed to benefit most.

In view of the MARCH data regarding the type of chemotherapy, most positive trials combined radiotherapy with three cycles of concurrent cisplatin 100 mg/m^2 ^[1] which can be considered the standard regimen. Budach et al. revealed the highest prolongation of survival of 24 months in combining 5-FU and radiotherapy [2]. Cisplatin- and carboplatin-based chemotherapy and radiotherapy prolonged the survival to 16.8 and 6.7 months, respectively.

However, it is a clinical fact that a significant percentage of patients do not receive their full planned course of combination treatment due to excessive toxicity, hence the need arises to optimize these regimen. Various options exist: first of all, the use of more tolerable chemotherapy combinations, second the integration of molecular targeted drugs and third the use of modern concepts of radiotherapy.

Staar et al. combined carboplatin with 5-FU and hyperfractionated accelerated radiotherapy in a randomized phase III trial [4] and presented comparable results to studies based on cisplatin. The published acute and late toxicity was moderate in this trial.

Another option of a treatment combination is the addition of targeted therapy approaches e.g. antibodies or small molecules. Squamous cell carcinomas of the oro-, hypopharynx and larynx often show an overexpression of epidermal growth factor receptors (EGFR), which is described to be associated with a poor prognosis [5-7].

Cetuximab is a monoclonal antibody binding to the extracellular EGFR domain. Intracellular phosphorylation of the EGFR is inhibited and consequently the down stream signalling is deficient resulting in cell cycle arrest and increased expression of pro-apoptotic enzymes. Further effects of EGFR-inhibition that have been already published are a reduction of cell proliferation and angiogenesis, as well as an increase of apoptosis [8,9]. Cetuximab has been found to potentiate the effects of chemotherapy and radiotherapy in experimental systems [8-10].

In a clinical setting, Bonner et al., in a pivotal phase III trial, compared radiotherapy alone vs. radiotherapy combined with cetuximab. In this trial, a statistically significant increased overall survival and local control rate could be found for the combined treatment regimen [11,12]. Furthermore, beside skin reactions and a slight increase of infusion reaction no further severe side effects were reported. Apart from these medication specific side effects, no significantly increased toxicity caused by the combination of radiotherapy and cetuximab could be found. This study was the first to demonstrate in a clinical phase III setting that the combination of radiotherapy and a monoclonal antibody against the EGF-receptor resulted in a clear survival benefit. When comparing the results of the Bonner study with chemoradiotherapy studies, it can be assumed that comparable results could be achieved with this new combination. In a retrospective literature review of chemoradiation data, the Bonner regimen yielded comparable results, hence this treatment is an alternative for patients who might not complete standard therapy due to co-morbidities. Unfortunately though, there is no phase III trial evaluating RCT vs. RT + cetuximab yet.

A further possibility to improve the therapeutic ratio is the integration of modern techniques of radiotherapy in current treatment schedules. Techniques like intensity modulated radiotherapy (IMRT) can reduce the acute and late toxicity and allow, through better protection of the surrounding organs at risk, the application of higher doses without increased toxicity and higher conformity.

The REACH trial is a phase II trial evaluating the combination of modern radiotherapy techniques (IMRT) and standard chemotherapy with the EGFR-inhibitor cetuximab. The fractionation regimen applied for IMRT corresponds to the established accelerated-hyperfractionated regimen in conventional 3D-RT, hence the same doses per fraction and total dose are prescribed. Therefore, we would assume no increased risk concerning RT with this modern technique. It seems that IMRT in this specific set-up can even further reduce potential side effects. Hence, better local control rates could be achieved due to the advantages of IMRT in receiving a better dose distribution in the target volume.

## Methods/Design

### Trial organization/coordination

REACH is a single-treatment group, bi-centric trial designed by the study initiators of the Department of Radiation Oncology of the University of Heidelberg. In order to accelerate the data collection, the Helios Klinikum of Berlin Buch will also participate in this clinical trial. The trial is carried out by the Department of Radiation Oncology of the University of Heidelberg which is therefore responsible for overall trial management, trial registration (ClinicalTrials.gov Identifier: ISRCTN87356938), database management, quality assurance including monitoring and reporting. The trial is an investigator initiated trial (IIT). Trial medication cetuximab (Erbitux^®^) is supplied by Merck KGaA, Darmstadt, Germany.

### Investigators

Patients will be recruited by the Departments of Radiation Oncology at the University of Heidelberg and the Helios Klinikum of Berlin-Buch. Due to the multi-modal nature of the trial, all investigators are experienced oncologists in the fields of radiation oncology and medical oncology.

### Quality assurance

According to Good Clinical Practice (GCP) and other applicable guidelines and regulations the side monitoring will be carried out by an independent monitor contracted by the sponsor.

### Ethics, informed consent and safety

The final protocol was approved by the ethics committee of the Medical Faculty Heidelberg (AFmu-005/2009), the local ethics committee Berlin and the Paul-Ehrlich-Institute (PEI-registration number 789/01). This study complies with the Declaration of Helsinki in its recent German version, the Medical Association's professional code of conduct, principles of Good Clinical Practice (GCP) guidelines and the Federal Data Protection Act. The trial will also be carried out adhering to local legal and regulatory requirements.

Written informed consent is obtained from each patient in oral and written form before inclusion in the trial. Nature, scope and possible consequences of which will been explained by a physician. The investigator will not undertake any measures specifically required for the clinical trial until valid consent has been obtained.

### Study design

The REACH study is a prospective phase II study combining the monoclonal EGF-receptor antibody cetuximab (Erbitux^®^) with standard chemotherapy and loco-regional irradiation therapy as intensity-modulated radiation therapy (IMRT) in patients with locally advanced squamous-cell carcinomas of oropharynx, hypopharynx or larynx. Patients will be treated by the Department of Radiation Oncology and Radiation Therapy, University of Heidelberg, in co-operation with the Helios Klinikum Berlin-Buch.

The primary endpoint of the study is the local-regional control (LRC).

The secondary endpoints are disease-free survival (DFS), progression-free survival (PFS), overall survival (OS), acute and late radiation effects, adverse events, proteomics and genomics.

### Patient selection

A total of 60 subjects with locally advanced, previously untreated, primary non-metastatic, squamous cell carcinoma located in the oro-, hypopharynx or larynx will be included. Each subject will receive the trimodal treatment as described above [radiation therapy (IMRT), chemotherapy (carboplatin and 5-FU) and cetuximab].

Inclusion criteria:

• Signed written informed consent

• Age between 18 and 70 years

• Life expectancy > 6 months

• Histologically confirmed locally advanced (stage III or IV), non-metastatic squamous cell carcinoma of oro-, hypopharynx and larynx (T_2-4_, N_x_, M_0_)

• Oral cavity or oro-, or hypopharynx or larynx as the primary tumor site

• At least one uni-measurable lesion according to RECIST criteria

• Karnofsky Performance Status > 70%

• Adequate bone marrow, liver and renal function: wbc > 1.5 × 10^9^/l, THC > 100 × 10^9^/l, hb > 10.0 g/dl, Bili < 2.0 g/dl, SGOT, SGPT, AP, GGT < 3 × ULN, sCrea < 1.5 mg/dl

• Commitment to use of adequate contraception

Exclusion criteria:

• Previous chemotherapy, radiotherapy or surgery for carcinoma of the head and neck

• Nasopharyngeal carcinoma

• Prior exposure to EGFR pathway targeting therapy

• Patients with unstable cardiac disease despite treatment, congestive heart failure NYHA III/IV, significant neurological or psychiatric disorders including dementia or seizures, active disseminated intravascular coagulation, symptomatic peripheral neuropathy Common Toxicity Criteria (CTC) grade II or higher as well as ototoxicity CTC II or higher except if due to trauma or mechanical impairment due to tumor mass.

• Pregnant or breast-feeding women

• Known allergic/hypersensitivity reaction to any drugs scheduled for the study treatment

• Participation in other interventional trial within the last 30 days

• Surgery within the last 30 days

• Known drug abuse

• Other previous malignancy within 5 years, with exception of a history of a previous, adequately treated basal cell carcinoma of the skin or pre-invasive carcinoma of the cervix

### Work-up

Pathologically documented squamous cell oro-, hypopharynx or larynx carcinomas will be sent for surgical consultation. Patients receive a complete work-up including examination under anaesthesia, head and neck CT scans, chest x-ray, abdominal ultrasound and bone scan. In case operation is either surgically or medically impossible or the patient refuses to undergo the procedure, in- and exclusion criteria will be examined and eligibility will be analyzed. Should a patient meet the trial conditions, information about participation in the study including potential risks and benefits is given to the patient. As soon as written consent is obtained, patients can be included into the trial and the required documentation will be provided by the study centre (Studienzentrale Klinische Radiologie, Abt. Strahlentherapie und Radioonkologie, INF 400. 69120 Heidelberg).

After inclusion, each patient receives a RT-planning CT-scan in an individually-adjusted precision immobilisation devices.

If a patient refuses treatment within the REACH trial, standard combined chemoradiotherapy (Carboplatin/5-FU, without cetuximab) will be offered.

### Safety and discontinuation of treatment

Toxicities are classified by type and grade due to NCI CTCAE v. 3.0, furthermore by duration, onset, and relationship to study treatment. Treatment of cetuximab-induced adverse reactions is carried according to recommendations by the manufacturer [13].

For grade 1 or grade 2 allergic reactions, a decrease of infusion rate for current and subsequent infusions is suggested. For ≥ grade 3, persistent grade 1 or grade 2 allergic reactions despite reduction of infusion rate, it is recommended to discontinue treatment with cetuximab. Skin reactions in terms of acne-like rash after cetuximab are common. For patients facing a grade 3 acne-like rash, cetuximab should be delayed for up to two subsequent infusions. Treatment also includes concomitant topical and/or oral antibiotics where necessary. Therapy can be resumed on resolution of the rash to < grade 2. Cetuximab needs to be delayed on a second or third occurrence of a grade 3 skin reaction for up to two consecutive cycles with dose reduction to 200 mg/m^2 ^or 150 mg/m^2 ^respectively. Any further occurrence of grade 3 acne-like rash will lead to discontinuation of cetuximab treatment according to the recommendations of the summary of product characteristics.

### Drug supply

#### Cetuximab (Erbitux^®^)

The monoclonal antibody cetuximab (Erbitux^®^) is provided by Merck KGaA, Darmstadt, Germany, and is stored by the University Hospital Pharmacy, Heidelberg.

**Figure 1:**
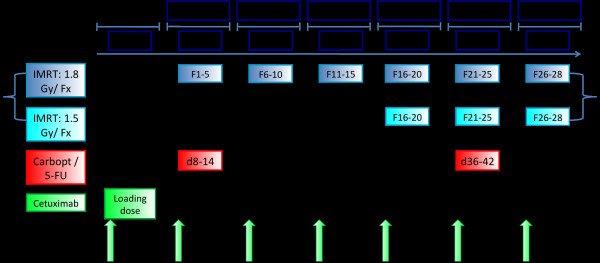
**REACH treatment schedule: Cetuximab (Erbitux^®^) is given at a loading dose of 400 mg/m^2 ^body surface on day 1, the current dose during radiotherapy on days 8, 15, 22, 29, 36 and 43 is 250 mg/m^2 ^body surface**. Chemotherapy (Carboplatin 70 mg/m^2 ^body surface/5-FU 600 mg/m^2 ^body surface) is given on days 8-12 and 36-40. Radiotherapy (IMRT) starts one week after the loading dose of cetuximab an day 8. From day 29 onwards, an additional fraction of RT (concomitant boost) will be given to a total dose of 69,9 Gy.

The cetuximab dose applied in this setting corresponds to the recommended and approved dosage tested in combination with irinotecan in metastatic colorectal carcinoma [14]. Cetuximab is given with a loading dose of 400 mg/m^2 ^of body surface as an intravenous infusion on day 1. Subsequently, the regular weekly dose during the radiotherapy is 250 mg/m^2 ^of body surface on days 8, 15, 22, 29, 36, 43 (Figure 1). A prophylactic premedication with corticosteroids and antihistamines is required to reduce the incidence of infusion-related reactions such as allergic/hypersensitivity reactions [13].

#### Chemotherapy

Carboplatin will be applied at a dose of 70 mg/m^2 ^body surface as a one-hour intravenous infusion on days 8-12 and 36-40 (corresponds to RT- week 1 and week 5 of RT, Figure 1).

5-FU will be applied at a dose of 600 mg/m^2 ^of body surface as an intravenous infusion over 24 hours on days 8-12 and 36-40.

#### Radiation Therapy

Irradiation is applied as intensity-modulated radiation therapy (IMRT) using either a 6 MV linear accelerator (Siemens) with motoric multi-leaf collimator in a step and shoot technique or a helical tomotherapy machine (Tomotherapy, 6 MV). The primary tumor and cervical lymph nodes receive a dose of 50,4 Gy in daily fractions of 1.8 Gy (Monday to Friday). From study day 29 onwards, patients receive an additional fraction at 1.5 Gy per day (concomitant boost) to a total dose of 69.9 Gy (Figure 1). There is at least a 6-h interval between the two daily fractions. Isocentre and patient positioning are checked on the first fraction and then at least weekly by CT. Radiation therapy can be carried out on an out-patient basis apart from days 8-12 and 36-40 (chemotherapy), unless the patient's condition requires hospital admission.

### Target Volumes and dose prescription

The primary tumor and involved nodes - based on clinical and endoscopic findings - constitute the gross tumor volume (GTV). Nodes are considered positive if larger than 1 cm in diameter or showing a necrotic centre. The CTV contains the GTV and areas of potential microscopic spread. CTV1 consists of the primary/positive lymph nodes (GTV) and a margin of approx. 1 cm, CTV2 includes the GTV with a margin of about 2 cm as well as the lymph node areas for elective nodal irradiation. The planning target volume (PTV) also accounts for set-up variations and internal organ motion, hence the CTV is usually expanded by a margin of >0.5 cm. In cases where critical organs are directly adjacent to either GTV or CTV, margins will, of course, be adjusted. Doses are prescribed to the respective PTVs: PTVHD (consisting of CTV1 plus margin) is prescribed a total dose of 69,9 Gy whereas PTVLD (CTV2 plus margin) should receive a total dose of 50.4 Gy.

The PTVHD will at least be covered by the 95% prescription isodose; in addition, no more than 20% of the PTVHD should receive ≥ 110% and no more than 3% of any part of the PTVHD or PTVLD should receive ≤ 90% of the prescribed dose.

Doses of >110% of prescribed dose outside the target volume should be limited to <1 cm^3^.

For critical normal structures a margin of 0.5-1 cm around the spinal cord may be added to create a Planning Organ at Risk Volume (PRV). The dose to any point within the spinal cord should not exceed 45 Gy to any volume larger than 0.03 cm^3^. The maximum doses of 54 Gy to the brain stem are accepted. However, if absolutely necessary, doses of max. 60 Gy can be tolerated to brain stem volumes < 60 Gy.

The mean dose to the parotid gland should be limited to <26 Gy, alternatively at least 20 cc of the combined volume of both parotid glands to < 20 Gy or at least 50% of one gland to <30 Gy. Whenever feasible, dose to the larynx should be kept below 45 Gy.

The dose to the brachial plexus must be limited to ≤ 60 Gy in patients with suspicious level IV node(s).

Mandible: 70 Gy should not be exceeded at any point.

### Supportive Therapy

Antihistamines such as clemastine or dimentinden and steroids are administered intravenously prior to cetuximab-application. Skin reactions, especially acne-like rashes can be treated by topic or systemic antibiotics (i.e. tetracyclines, metronidazole or nadifloxacine) if necessary.

Whenever it is necessary, metoclopramide or 5-HT_3_-antagonists are used for antiemesis.

Radiation induced skin reactions are treated according to in-house protocols with mild moisturizing lotion or local application of steroids.

### Blood Samples

It is planned to collect approximately 300 ml of blood from each study subject during his/her trial participation, i.e. within a period of maximum 60 consecutive months. Potential risks of blood samplings are well predictable and include rare and mostly mild complications such as vascular injury, reversible nerve irritation and/or bleeding. Considering a potential gain of relevant information on genomic/proteomic mechanisms and predictive marker for clinical outcome in patients with advanced cancer of the head and neck, blood sample collection during the trial seems to be highly justifiable.

### Adverse events

Radiotherapy-related toxicities will be assessed using the NCI Common Toxicity Criteria (CTCAE v.3.0). Toxicity will be evaluated at baseline, weekly during radiation therapy (blood count, electrolytes, chemistry, clinical examination, patient visits) and at follow-up visits. Unacceptable toxicity is defined as unpredictable or irreversible grade 4 toxicity.

Expectable possible acute toxicities (up to 3 months after irradiation) comprise skin toxicity (desquamation, erythema, hyperpigmentation), nausea, vomiting, fatigue, weight loss, loss of appetite, pneumonitis, haematological toxicity with leucozytopenia, thrombozytopenia or anaemia. These symptoms could be treated medically and therefore usually resolve within 2-3 weeks. However, transient parenteral nutrition and hydration might be necessary in some cases. All acute toxicities should completely resolve within a few weeks post radiation therapy.

Decisions regarding cetuximab as well as chemotherapy dose adjustment will be made by using the guidelines below and are based on haematological parameters (ANC and platelets) monitored weekly during radiation before each dose of cetuximab and chemotherapy.

Expedited reporting will be carried out according to the local regulations.

### Evaluation

Local response is evaluated in accordance with the RECIST criteria (Response Evaluation Criteria in Solid Tumours) [15].

• Complete remission (CR) is defined as complete regression of the treated tumor mass (confirmation after at least 4 weeks of treatment or later)

• Partial remission (PR) is defined as reduction of sum of largest tumor diameters by at least 30%

• Stable disease (NC = no change) is defined as neither PR nor PD

• Progressive disease (PD) is defined as increase of sum of largest tumor diameters by 20%

### Sample Size Calculation

The choice of number of patients is based on pragmatic reasons. Therefore, the sample size analysis is replaced by a power analysis giving the expected accuracy of the results.

It is estimated that the two-year Local Regional Control rate will be 75 per cent. Given a constant hazard rate over time (leading to an exponential distribution), and assuming a two-year accrual time and a three-year follow-up time (leading to a median observation time of less than four years), with 60 patients treated the three-year LRC rate can be estimated with a 95%-confidence interval of approximate width of 18 per cent, i.e. if the LRC estimate is 65 per cent, the limits can be expected to be at 53 and 81 per cent.

### Statistical Methods

The time to event for local regional control, disease-free survival, progression-free survival, overall survival will be calculated using a Kaplan-Meier estimate, along with a 95 per cent confidence interval.

Acute and late radiation effects as well as adverse events will be tabulated and listed by seriousness, severity, System Organ Class and relatedness.

Biometric analysis will be specified in more detail in the statistical analysis plan which has to be authorized before opening the database for analysis by the biometrician, the sponsor, and the LKP.

## Discussion

Assuming that intensification of established chemoradiotherapy by the addition of cetuximab can further improve outcome, the primary end point of this study is local regional control. Treatment-related side effects should not be increased as compared to standard combined chemoradiotherapy. As already discussed, the combination of radiotherapy and cetuximab has shown promising results with only marginally increased toxicity or risks. In comparison with chemotherapeutic agents, treatment with EGFR inhibitors is associated with lower incidence of systemic side effects. Despite these benefits, there are some commonly occurring side effects of EGFR inhibitors (papulopustular rash, dry skin, itching, hair and periungual alterations), which can result in reduced quality of life as well as a reduction, interruption or discontinuation of cetuximab treatment. An evaluation of acute toxicity of skin and mucosa in patients with head and neck cancer receiving radiotherapy (RT) alone or in combination with radiotherapy plus chemotherapy (RCT) or with cetuximab (RIT) showed a grade 3 toxicity of the skin in 27,6% of the RIT patients (vs. 0% of RT, 7% of CRT) [16]. Typical appearance of grade 3 skin toxicity in RIT was a massive confluent desquamation of the RT field. Acute grade 3 mucositis was observed in 24,6% of RIT (vs. 12,5% of RT, 12,5% of RCT). Cetuximab associated acneiform rash grade 3 was observed in 7% of the RIT patients. Eight weeks after RT, all patients had recovered from these side effects. Cetuximab did not lead to a higher rate of RT interruptions compared to RT or RCT. Budach et al. reported on two patients with squamous-cell carcinoma of the head and neck who had severe radiation dermatitis while receiving a combination of radiotherapy and cetuximab [17]. Severe radiation dermatitis may occur after irradiation alone, but grade 4 lesions are rarely observed. Coexisting conditions like previously received chemotherapy or radiotherapy, liver or renal dysfunction with possible alterations of pharmacodynamics of cetuximab, may predispose patients to the development of more severe radiation dermatitis [17]. Another trial reported that 49% of patients treated with cetuximab and concurrent radiotherapy developed a grade III or IV radiation dermatitis [18]. Hence, the incidence of these severe skin reactions was twice as high compared to that reported by Bonner et al. [11]. To evaluate the real percentages of grade III and IV radiation dermatitis in the treatment of cetuximab with concurrent radiotherapy, a prospective, large-scaled study is needed. Currently, the exact mechanism causing these enhanced skin reactions remains to be clarified. With collecting blood samples, we try to find strategies for the identification of phenotypes and genotypes that are at risk of skin toxicities.

Chemotherapies like cisplatin or 5-FU combined with cetuximab have been used in the treatment of recurrent and/or metastatic squamous cell carcinoma of the head and neck. The results are promising and no increased or overlapping toxicity was found. In the primary treatment of locally advanced squamous cell carcinomas of head and neck, combination of chemoradiotherapy and cetuximab resulted in increased acute side effects in a phase II trial with 22 patients [6]. In the study, marked adverse events occurred, including two deaths (one pneumonia and one unknown cause), one myocardial infarction, one bacteremia, and one atrial fibrillation. With a median follow-up of 52 months, the 3-year overall survival rate was 76%, the 3-year progression-free survival rate 56%, and the 3-year locoregional control rate 71%. Despite the positive clinical results, the trial was closed prematurely. An investigation revealed no evidence that the adverse events could be attributed to the addition of cetuximab. Recently, the Radiation Therapy Oncology Group (RTOG) initiated a phase II trial [19] based on the protocol of Pfister et al. [20]. Concerning this proposed trial, no increased risk or toxicity seemed evident and therefore the benefit for the patients justifies conducting the trial. Clear instructions are defined in the protocol to ensure the safety of the patients.

Xerostomia is the most common late toxicity of radiotherapy to the head and neck. IMRT can reduce the dose delivered to the parotid glands. An analysis of Nutting et al. showed reduction of xerostomia ≥G2 (LENT-SOMA scale) at 12 and 18 months post radiation therapy from 74% (CRT) to 40% (IMRT) and 71% (CRT) and 29% (IMRT) respectively [21]. A retrospective study of Clavel et al. compared toxicity and efficacy of CRT and IMRT treated with concomitant chemotherapy (carboplatin and 5-FU) for locally advanced oropharyngeal cancer [22]. At a median follow-up of 33 months OS, DFS and LCR were significantly higher for IMRT. Additionally, patients treated with IMRT had less dermatitis and xerostomia at 24 and 36 months.

## Competing interests

The authors declare that they have no financial or non-financial competing interests. However, the trial medication (cetuximab, Erbitux^®^) and a financial grant for study organisation are supplied by Merck KGaA, Frankfurter Str. 250, 64293 Darmstadt, Germany.

## Authors' contributions

GH, ADJ, MWM, RK and JD planned and co-ordinate the study. GH, ADJ, MWM, RK, KP, HH, MU, CS and JD are conducting the study. Medical care is covered by GH, ADJ, KP, CS, MU, HH and MWM. MWM and RK are responsible for the patient recruitment. GH, ADJ, RK, MU, HH and MWM perform planning and radiation therapy. All authors read and approved the final manuscript.

## Pre-publication history

The pre-publication history for this paper can be accessed here:

http://www.biomedcentral.com/1471-2407/10/651/prepub
